# Simulated deep space exposure on seeds utilizing the MISSE flight facility

**DOI:** 10.1038/s41526-024-00451-y

**Published:** 2025-01-17

**Authors:** Jeffrey T. Richards, Todd E. Mortenson, Cory J. Spern, Timothy A. Mousseau, Jennifer L. Gooden, Lashelle E. Spencer, Christina L. Khodadad, Jason A. Fischer, Alexander D. Meyers, Chad K. Papenfuhs, Jeffrey G. Buell, Howard G. Levine, Dinah I. Dimapilis, Ye Zhang

**Affiliations:** 1 NASA John F. Kennedy Space Center, Kennedy Space Center, Merritt Island, FL USA; 2 AETOS Systems Inc., LASSO II Contract, Kennedy Space Center, Merritt Island, FL USA; 3 The Bionetics Corporation, LASSO Contract, Kennedy Space Center, Merritt Island, FL USA; 4 Noetic Strategies, Inc., LASSO II Contract, Kennedy Space Center, Merritt Island, FL USA; 5 Department of Biological Sciences, University of South Carolina, Columbia, SC USA; 6 NASA Postdoctoral Program, John F. Kennedy Space Center, Kennedy Space Center, Merritt Island, FL USA; 7 NASA Internship Program, John F. Kennedy Space Center, Kennedy Space Center, Merritt Island, FL USA; 8 Aegis Aerospace Inc, Houston, TX USA

**Keywords:** Plant sciences, Biotechnology

## Abstract

The MISSE-Seed project was designed to investigate the effects of space exposure on seed quality and storage. The project tested the Multipurpose Materials International Space Station Experiment—Flight Facility (MISSE-FF) hardware as a platform for exposing biological samples to the space environment outside the International Space Station (ISS). Furthermore, it evaluated the capability of a newly designed passive sample containment canister as a suitable exposure unit for biological samples for preserving their vigor while exposing to the space environment to study multi-stressor effects. The experiment was launched to the ISS on Northrup Grumman (NG)-15. The exposure lasted eight months outside the ISS in the MISSE-FF at the Zenith position. The specimens consisted of eleven seed varieties. Temperature dataloggers and thermoluminescent dosimeters were included in each container to record environmental data. We presented here the hardware and experimental design, environmental profiles, and seed survival from post-flight germination tests.

## Introduction

With the renewed goal of crewed Mars exploration, continuous fresh food production during long-term deep space missions will be a critical addition to the packaged food system to meet astronauts’ nutritional requirements and to provide a psychological countermeasure for the crew in the isolation and confinement of deep space. However, critical knowledge gaps, such as the impact of deep space radiation on long-term seed viability and plant growth, must be addressed prior to dependence on crop systems for any portion of a deep space food system. These knowledge gaps are also relevant to future long duration missions and for the establishment of permanently inhabited planetary surface bases.

Investigations on the impact of long-duration deep space exposure on biological systems are limited by the availability of flight platforms and the limitation of ground-based analogs. There are several flight platforms capable of exposing biological samples to space-relevant environments. One of these is the previous NASA’s Materials International Space Station Experiment (MISSE), an orbiting commercial science and test facility permanently installed on the exterior of the International Space Station (ISS). Previous life sciences missions outside the ISS include MISSE-3 and -4, containing basil, radish, tomato seeds, and several microbes. In addition to MISSE, EXPOSE is a multi-user facility mounted outside the ISS deployed by the European Space Agency (ESA)^1–3^. EXPOSE -E -R, and -R2 missions have flown *Arabidopsis*, tomato, and tobacco seeds, as well as other organisms^4,5^. The harsh environment outside the ISS is relevant to deep space. Historical data showed temperature fluctuations from −27 °C to 46 °C during each 90 min orbital loop. Radiation exposures (with 0.6 g cm^−2^ shielding) were 330–469 μGy/day based on passive thermoluminescent dosimeters (TLD) readings. Active R3DR readings showed 506 μGy/day protons, 81.4 μGy/day Galactic Cosmic Rays (GCRs), and 89 μGy/day energetic electrons. Without shielding, UV (200–400 nm) fluence is high, at 834–1130 MJ m^−2^. In addition, without protection, samples are potentially exposed to space vacuum, atomic oxygen species, and other contaminants.

The primary goal of this MISSE-Seed project (part of the MISSE-14 mission) was to test new sample containment canisters and the interface with the current Multipurpose Materials International Space Station Experiment—Flight Facility (MISSE-FF) platform for exposing biological samples to the space environment. MISSE-FF provides NASA’s closest analog to the long duration deep space radiation environment with sample return capabilities. The true low-dose rate space radiation environment with a broad spectrum of charged particles experienced in MISSE-FF cannot be simulated on the ground. MISSE-FF can, therefore, support investigations on the long-term combined impact of deep space radiation and microgravity.

On February 20, 2021, the MISSE-Seed experiment was launched on Northrup Grumman (NG)-15 to the ISS. The science aimed to investigate the effects of space radiation on seed quality and storage. This project also validated newly developed passive sample containment canisters for providing acceptable storage conditions for seeds or other biological samples upon exposure to the space environment outside the ISS. A total of 11 seed types were packed within the canisters along with TLDs and temperature dataloggers. Seeds were exposed to the space environment outside the ISS for eight months and inside the ISS for about three months. After the sample return, preliminary post-flight analyses included seed germination rates and seedling development to determine the cross-species responses to the space environment exposure.

## Results

### Hardware development

MISSE passive exposure containers (MPECs) were developed and fabricated for housing the biological samples using 6061-T651 aluminum plates. Eight MPECs each were used for containing flight and ground control samples. Several fabricated containers were subject to thermal testing with 37 °C to −37 °C to 37 °C cycles (90 min per cycle) and different configurations of insulation materials using aerogel foam (0.5 mm Spaceloft, Aspen Aerogels), polyimide foam layers, and polyimide foam edging as insulating materials. The setup and results of these pre-flight tests are presented in the “Pre-flight validation test results” section.

**Fig. 1:**
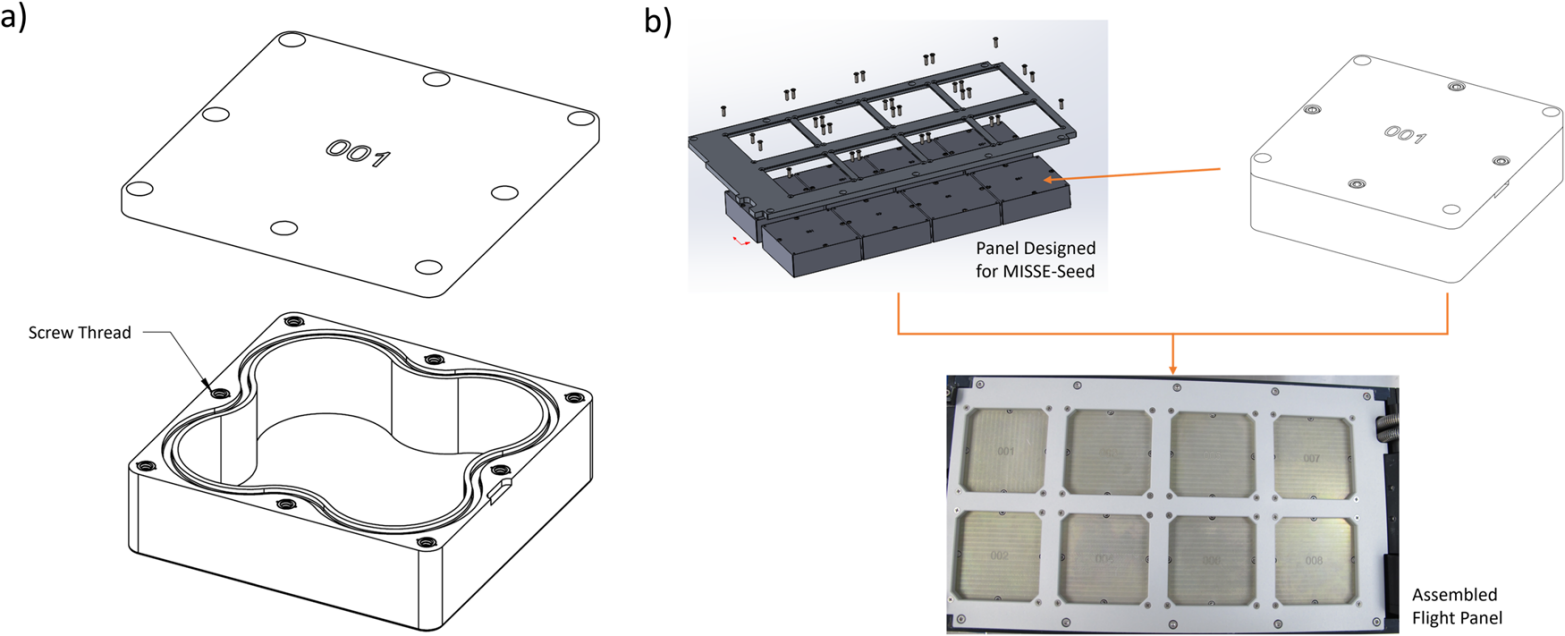
Designs of MPEC and customized MSC panel. **a** Drawing of an MPEC and **b** Customized MSC panel and the assembled flight panel.

Dimensions for each MPEC is 7.5 × 7.5 × 2.5 cm (Fig. 1). The thickness of the lid and the bottom is 2 mm. The side of the container is designed with curves to maximize the interior size and to allow required space for screws to assemble each individual container and to install MPECs to the MISSE-FF panel. A customized eight-slotted panel was designed by Aegis Aerospace to accommodate MPECs (Fig. 1). Four screws at the corner of the MPEC were for installing the MPEC to the MISSE Science Carrier (MSC) panel, and the other four screws were to assemble the container with samples. All surfaces of the boxes were alodined prior to use. Numbers were etched on the lids for MPEC identification. A groove was machined into each base for placing an O-ring to seal each container, and a small gap area was available for tool insertion for opening the MPEC’s more easily. To assemble the containers, a layer of RTV sealant was applied around the edge of the O-ring before closing the lid and securing the container with screws. Multiple sealant materials were evaluated for biocompatibility, and the results are presented in the “Pre-flight validation test results” section.

### Pre-flight validation test results

#### Seed storage temperature test

**Fig. 2:**
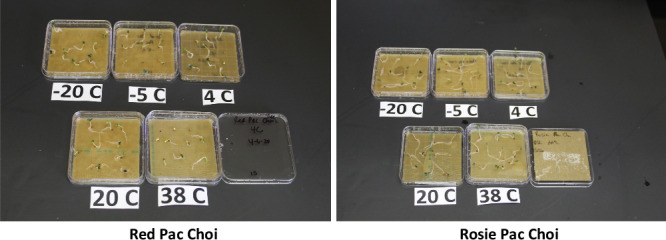
Examples of germination tests of crop seeds after 21-day storage at different temperature conditions. Photos were taken at Day 4 after the initiation of the test for red pac choi seeds (left) and Rosie pac choi seeds (right) for analysis.

In addition to *Arabidopsis thaliana* (Col-0), a cohort of 31 crop seed types was initially selected for this study. After storage under five temperature conditions at −20 °C, −5 °C, 4 °C, 20 °C, or 38 °C for 21-day durations, seeds were evaluated for germination viability. Signs of poor health, such as discoloration, growth abnormalities, and slow development, were noted. The diverse seed types responded to unfavorable storage temperature challenges differently, even within the same species (Table 1). For example, Rosie pak choi seeds showed better tolerance to 38 °C than red hybrid pak choi and were therefore chosen for the flight experiment (Fig. 2). Seeds were grouped into one of four categories based on their temperature test performance: (A) Seeds with germination rate equal or higher than 80% under all the conditions; (B) seeds showing lower germination rate (<80%) under one or both extreme temperature conditions (−20 °C and/or 37 °C); (C) seeds showing lower than 80% germination rate under moderate temperature ranges (−5 °C to 20 °C); and (D) seed germination rate equal or lower than 50% in all conditions. The poor germination rate for the groups C and D seeds may be caused by many reasons including seed type (e.g., the challenge of analyzing Swiss chard seed germination rate) and experimental conditions (light level, growth temperature, and nutrient supply). Among the 32 seed types assessed, 11 were chosen for the flight experiment.

**Table 1:** Germination rate of seeds before down-selection to 11 varieties (shown highlighted in green) after 21-day storage at different temperature conditions

Ten seeds per seed type were used for each test condition except for Arabidopsis *tested with more seeds.*

#### Thermal cycle test

A thermal cycle test was conducted to provide baseline data on the thermal protection capabilities of MPECs using several configurations of Spaceloft and Polyimide foam insulation layers. MPEC test units were exposed to thermal cycles that mimicked the estimated external space environment for MISSE-14 using the KSC Engineering Development Lab (EDL).

**Fig. 3:**
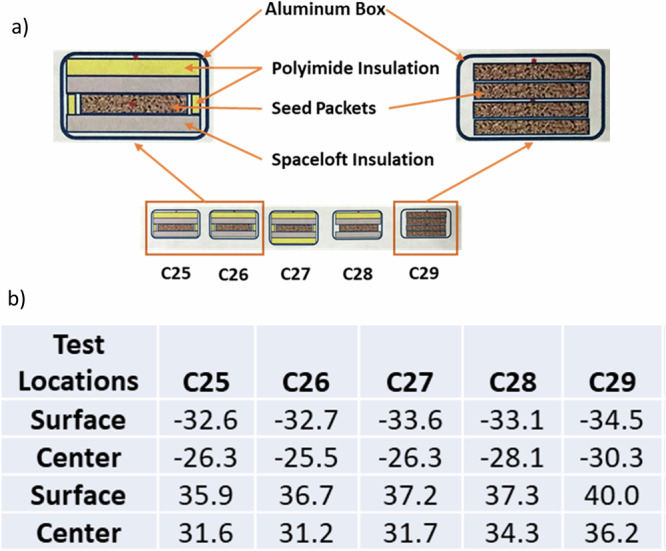
Thermal test configurations and maximum temperature recorded. **a** Different configurations of MPEC assembly, including C25 and C26 (two Spaceloft layers, one polyimide layer on top of one of the Spaceloft layers, and one polyimide layer at the side of MPEC), C27 (two Spaceloft layers with two polyimide layers, and one polyimide layer at the side of MPEC), C28 (two Spaceloft layers and one polyimide layer on top of one of the Spaceloft layers without side insulation), and C29 (no insulation materials) and **b** Maximum temperatures (°C) recorded right underneath the lid and at the center between seed packets from each individual MPEC configuration.

Five test units were fabricated identical to the flight MPECs except for a hole on each to pass through two thermocouples per container (one right underneath the lid and the other in the middle of the container wrapped by layers of insulation materials and/or seed packets). Twelve seed pouches were placed in each box. Spaceloft® aerogel insulation layers, polyimide foam layers, and/or polyimide foam edging were placed into each test MPEC (Fig. 3). One to two programmed iButton temperature recorders were placed in each container. The test MPECs were placed in a thermal test chamber (Sun Electronic Systems, Titusville, FL 32780) and subjected to four 90 min cycles of −37 °C to 37 °C to −37 °C.

Not surprisingly, the test MPECs without any insulation materials experienced the most extreme temperature fluctuations inside the containers (Fig. 3b, C29). Even with the insulation materials, without the polyimide foam edging material to protect the interior packets (Fig. 3b, C28) from being exposed directly to the surface of the container, the inside temperatures were harsher than those in the container with both insulation layers and edging materials (Fig. 3b, C25–C27). Although the protection of insulation materials on potential extreme temperature fluctuations may not be adequate to protect temperature-sensitive biological samples, the C25 and 26 configurations (with a polyimide and Spaceloft layer on top, a Spaceloft layer at the bottom, and edged with a polyimide layer) was deemed adequate for seed samples.

#### Sealant toxicity test

To evaluate the effect of the off-gassing of sealant materials on seeds, a biocompatibility test was conducted using lettuce and *Arabidopsis* seeds. Seed germination tests, with varying quantities of DowSil RTV3140, 3145 (Dow, Calgary AB, CAN), Siltrust RTV566 (Momentiv, Niskayuna, NY USA), MasterSil 921-Lo and 920-Lo (Masterbond, Hackensack, NJ, USA) were performed. Sealant materials were applied on the cover inside of a Petri plate while seeds were placed on wetted germination paper. Based on the results (not shown), RTV3140 was selected because of its low impact on seed germination.

#### Vacuum test

Stand-alone iButton temperature loggers and seeds sealed within mylar foil bags were placed under vacuum conditions to ensure the seed packets could sustain space vacuum or reduced pressure if any containers leaked, and that iButtons would still function as designed. After 5.5-hr exposure to vacuum conditions, iButtons retained full functionality to upload programs and download data, did not experience any material deformations, and seed packets remained intact.

### Flight environmental conditions

#### Radiation, UV, and atomic oxygen level measurements

Over the course of this experiment, total radiation doses measured by the TLDs in the MPECs varied from 272.69 to 340.54 mSv (mean = 305.00 ± 3.32 mSv). The mean total doses recorded for three different types of TLDs (TLD 100, 600, and 700 chips) ranged from 294.30 to 315.05 mSv. The average daily dose rate throughout this ISS mission while in space (including three months inside the ISS and eight months outside the ISS) was approximately 902.7 µSv/d. Calculated based on an estimated total radiation exposure dose of 100 mSv for a 6-month stay on the ISS, the measurements of TLDs outside the ISS in the MISSE-FF for this study may reach an average daily dose of 1062.5 µSv/d compared to the estimated dose of 555 µSv/d inside the ISS^6^. TLDs placed in the ground control MPECs showed a mean daily dose of 8.72 µSv. Dosimeters were calibrated in the NASA Space Research Lab (NSRL) at the Brookhaven National Lab (BNL) using SPEsim exposure and dose corrections were applied to all TLDs, although this may not be appropriate for the ground control TLDs where gamma rays were the main energy source.

The measurement provided by Aegis Aerospace for external ionizing radiation dose was 119.95 mGy (~204 mSv based on a quality factor of 1.7, about 1132.8 µSv/d) from 6/30/21 to 12/26/21 using RADFET dosimeters. This result is consistent with our interior measurement at about 305 mSv for the whole experiment period with an estimated average dose of 1062.5 µSv/d outside the ISS. The estimated total Å of other contaminants was 0.37, which should not affect the biological samples.

#### Temperature profile

**Fig. 4:**
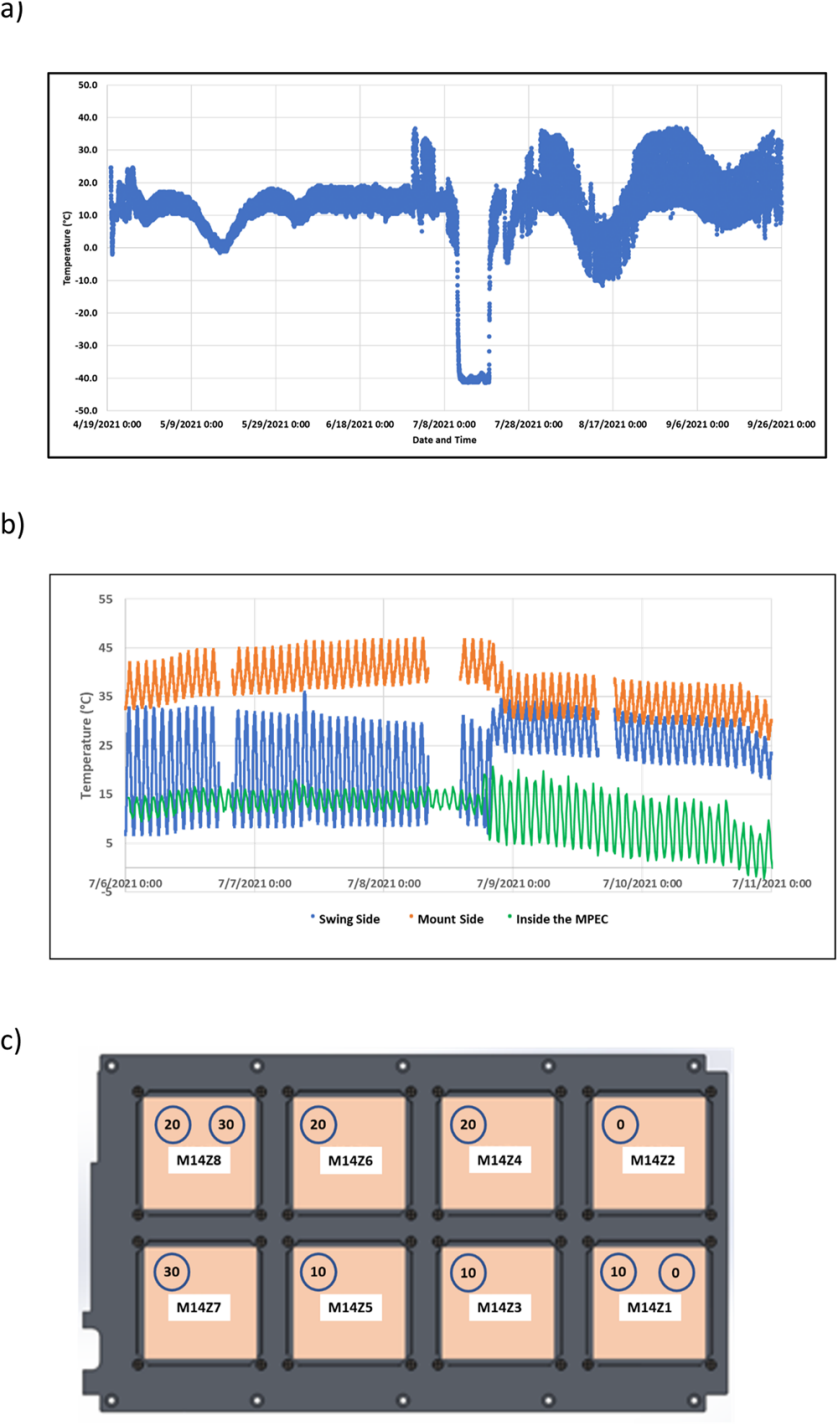
Recorded temperature profiles during the flight mission. **a** Temperature profile inside the MPEC sample containers (note that iButtons are only able to record to −40 °C so the extent of the temperature drop may be lower than indicated). **b** A section of data comparing the differences among the swing side, the mount side, and inside the MPECs (note the cyclic pattern of temperature recorded every 90 min throughout the orbits of the ISS). **c** Time-lapse settings of each iButton data recorder in 8 MPECs (M14Z1 to M14Z8) to start recording from time 0, or at 10 min, 20 min, or 30 min delay indicated in each circle representing each iButton.

During the space exposure outside the ISS (from 4/19/21 to 09/26/22) the average temperature inside the eight MPECs was 11.5 °C (ranging from −41.3 °C to 36.1 °C). Unfortunately, we were only able to obtain the interior profile with data loggers during this period because the data loggers were programmed to start recording data in February for 7 months. Due to launch/installation date delays, we only obtained the MPEC interior temperature data until 09/26/22. Aegis Aerospace provided the external temperature profile from 6/30/21 to 12/26/21. Based upon the differences between the interior and external profiles that were available, we extrapolated the missing data and generated the temperature profile for the entire mission (Fig. 4). Thermal profiles at different locations on the MSC (Swing side vs Mount side) and whether inside or outside the MPEC container were significantly different with generally milder temperature ranges at the swing side compared to the mount side. More controlled temperature ranges were recorded within the MPEC container.

### Post-flight germination test

**Fig. 5:**
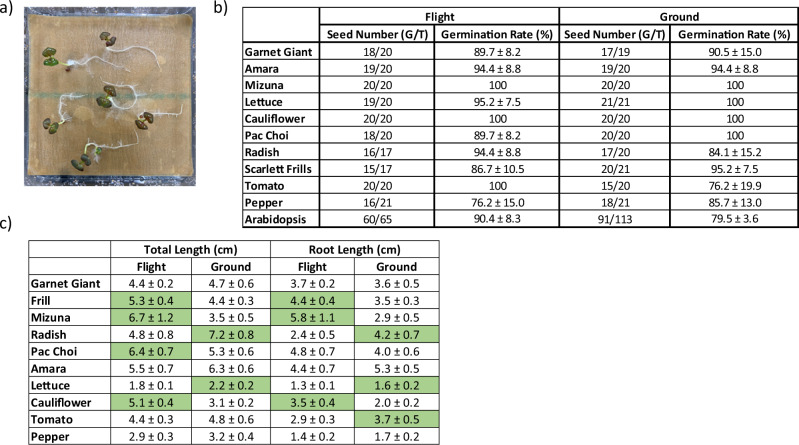
Post-flight germination evaluation. **a** Germination test setting. **b** Germination rate presented as the sum of the seed numbers from three replicate plates (germinated seeds [G]/total seeds [T]) and mean ± SEM in percentage from the same three plates calculated individually. No significant difference in germination rate was found between flight and ground seeds. **c** Seedling total length and root length presented as mean ± SEM in centimeters (cm) from all crop seedlings harvested from the same plates presented in (**b**). Significant differences at *p* < 0.05 between flight and ground seedlings are highlighted in green for the groups with significantly longer total or root length.

Germination tests were conducted for all seed types (Fig. 5). Germination rate, total plant length (root plus shoot), and root length from three replicate Petri plates were documented and analyzed. There were no significant changes in germination rate for all the seed varieties (Fig. 5b, presented in seed numbers and percentage), however significant changes in total length and root length were detected in seven seed types highlighted in green (Fig. 5c, presented in centimeters). The response was seed-type dependent.

## Discussion

This study used the MISSE-FF to investigate the effects of chronic exposure to cosmic radiation in combination with other space environmental factors on seeds. We purposely requested samples to be located at the Zenith location, which potentially has both sides of the MPEC facing into deep space, one side primarily to the Sun. This selection also avoids the potential exposure to direct reflection from the solar panels. It was anticipated that the position options within the MISSE-FF would significantly impact the environment the samples were exposed to, which needs to be considered for designing future studies. The impacts include (1) the ranges of temperature fluctuations throughout orbits and inclination of the ISS, (2) level of UV exposure, (3) levels of low energy particles, and (4) levels of high energy particles but to a much lesser extent. Based on the skin dose detected in the MATROSHKA phantom mission^7^, the seeds stored outside ISS could potentially receive 17 cGy, 34 cGy, 67 cGy, and 1 Gy in total dose for six-month, 1-year, 2-year, and 3-year exposures, respectively (equivalent to 300 mSv, 590 mSv, 1160 mSv, and 1770 mSv). Our radiation measurements were consistent with these predicted values as well as those from the EXPOSE-R missions. Even though the radiation characteristics in LEO differ from those in deep space, the radiation environment provides a broad spectrum of charged particles at a dose rate comparable to deep space with a quality factor of about 1.7.

Ideally a 3-year exposure on MISSE-FF would allow us to estimate the impact of a 3-year deep space mission. However, the current implementation model of MISSE-FF for each mission is from 6 months to 1 year. Therefore, this first platform validation mission targeted a six-month mission and lasted eight months. This mission’s focus was to evaluate space exposure’s impact on seeds, including both model organisms and crop plants, with a primary objective of testing whether the MISSE-FF and the newly configured MPECs and seed container placement within the MSC provide an acceptable exposure and experimental condition for biological samples.

The MISSE-Seed project timeline required a 6-month rapid turn-around development from when the opportunity was awarded by the Flight Opportunity Program to the science turnover. There were many accomplishments and lessons learned. Firstly, the design of MPEC and customized MSC panel was successfully flight validated. For the MPEC, a 1-in. depth could be easily increased to provide more interior space for the samples. Cautions are: (1) the mass could be increased significantly, and (2) the screw size currently used for securing the container itself and container to the panel may need to be reevaluated. Secondly, overall, the MPECs provided an acceptable thermal range for seed storage. The insulation materials likely attenuated the temperature fluctuations better in the lab setting with about 3–4 °C attenuation for the max temperature tested compared to the flight condition. This is mainly due to the difference between heat sources and heat transfer efficiencies. In general, the insulation materials applied in this study were not significantly effective. In contrast, the alodined surface with a potentially more reflective effect on the lid may contribute more to preventing excessive heat from solar light radiation. The two sides of the MPEC apparently experienced two thermal events, the top facing toward the Sun, and the bottom toward deep space. Therefore, the interior profile is significantly different from the external thermal profile for the sensors on the top of the panel. Thirdly, we realized that not only did the MSC position (e.g., zenith vs. nadir) provide different conditions to the science which could be critical, but the location of the experiment within the same MSC was critical too (e.g., swing side vs. mount side). Compared to the mount side, the swing side, where the MISSE-Seed experiment was situated, is relatively cooler with possibly larger swings in temperature within each cycle. This may be due to the direct contact of the mount side to the infrastructure that is connected to the ISS. Within the eight MPECs, there were no significant differences among the profiles recorded from different containers. Lastly, there always are environmental uncertainties during space exposure. There are some repeats of similar thermal cycles, however, with orbit changes, the thermal profiles change as well. Other events that could affect MISSE-FF thermal profiles dramatically include vehicle docking operations or orbits with high beta angles.

The second objective of this experiment was to evaluate the impact of space exposure on seed survival and quality. There have been several long-duration experiments exposing seeds (including *Arabidopsis*, rice, barley, lettuce, corn, arugula, and tomato seeds) to the space environment using different platforms such as the MISSE facility, the EXPOSE facility, space station MIR, ISS, and the long duration exposure facility (LDEF). Using the MISSE and EXPOSE facilities, seeds were exposed to the environment outside of the ISS. However, for the LDEF and MIR missions, seeds were stored inside which exposed them to an environment similar to the ISS. The results coming from these studies are not consistent, but many showed significant effects of space exposure. In a 27-month EXPOSE-R experiment stationed outside the ISS, analysis of the germination results revealed that the tomato seeds did not survive the exposure to radiation with or without UV. *Arabidopsis* seeds survived only when stored in the layer shielded from UV exposure and were able to germinate and further develop^8^. Tepfer et al. reported another EXPOSE-R experiment (without UV) showing a significantly reduced germination rate of seeds from two wild-type *Arabidopsis* lines: Columbia (Col-0) and Wassilewskija (Ws-2)^9^. Compared to Col-0, Ws-2 showed more sensitivity to space exposure. DNA degradation was found in the seeds exposed to UV, but not significantly in those shielded from UV exposure^9^. In another experiment, tomato seeds had been flown for 6 years on board the space station MIR. Hereditary variations were found in both the first and second generations of plants^10^. In contrast, under the LDEF flight condition, tomato seeds maintained at a controlled climate of 14 psi with 15% humidity at an altitude between 175 and 275 nautical miles only showed that the pores of the strophiole region of the flight seeds were larger in size than those in the ground control seeds, which might affect germination^11^. Interestingly, corn seeds exposed to the LDEF conditions showed damage to chloroplast development^12^.

Another study examined barley in a 13-month exposure to the environment outside the ISS, as well as rice in 13- and 20-month follow-up studies^13,14^. These seeds, which likely experienced similar radiation to the MISSE samples, displayed varying effects from space exposure. A 13-month exposure resulted in a 16% germination rate decrease in barley and a 48% decrease for rice. Rice seeds exposed for 20 months experienced a drastic 73% decrease in germination rate. Seeds that germinated successfully showed no significant changes in growth patterns or biomass for either rice or barley. In a different long-duration spaceflight experiment, seeds of arugula (rocket lettuce) were shielded within the space station for 6 months. Upon return to earth, these seeds exhibited delayed germination, but total germination rates were unaffected. Likewise, in the space-flown rocket seeds, growth and biomass were not altered compared to ground controls^15^. The species-specific effect of long-duration exposure to space radiation on plant seeds highlights the need for further experimentation in candidate food crops being considered to provide nutrition during extended human space travel. The platform developed for MISSE-Seed will help to facilitate future investigations in this area.

In addition to these flight opportunities, other space radiation-related research platforms have been utilized. In the Cosmic Ray Exposure Sequencing Science (CRESS) experiment using a 1U CubeSat system, *Arabidopsis* seeds were exposed to the stratosphere (36–40 km) environment above Antarctica in a 30-day, long-duration high-altitude balloon mission^16^. In a parallel experiment, *Arabidopsis* seeds were exposed to simulated galactic cosmic rays (GCR) in the NSRL. Both GCR and stratosphere-exposed seeds showed significantly reduced germination rates and significantly elevated somatic mutation rates with developmental aberrations^16^. In one of our radiation studies, various seeds were exposed to ground-based “simulated space radiation” exposures in the NSRL, including GCR and solar particle event (SPE). We confirmed for the first time that the exposures significantly affected the growth of *Arabidopsis* and Mizuna seeds. We observed the outcome was dependent on seed types and radiation quantity including dose, dose rate, and radiation quality, with heavier ions causing more severe damage^17^. These results are consistent with other published findings showing that the effects of ionizing radiation on plants are significantly influenced by species, cultivar, development stage, tissue architecture, and genome organization, as well as radiation features, e.g., quality, dose, and duration of exposure^18–21^.

In this MISSE-Seed study, post-flight we conducted an immediate germination test on all the seed types and compared germination rate, overall seedling length, and root length between seedlings from flight seeds and ground control seeds. The purpose of the test was to determine whether the environmental conditions experienced during the ISS flight experiment were acceptable for storage of seed samples following long-term space exposure and whether the space exposure has impacts on seed viability. Although there were differences in germination rates, they were not found to be statistically significant in this study. We do not, however, rule out possible significant changes where the number of seeds tested is greater. We found that the exposure promoted seedling growth within some species (Scarlett frills, mizuna, and cauliflower) and had inhibitory effects on others (radish, lettuce, and tomato). Among the ten crop seed types we flew, seven belong to *Brassica* family, also known as cole crops. All these types, except for radish, were not affected by space exposure or showed enhanced early seedling development observed in the immediate post-flight test. Many cole crops are hardy, especially in cold climates, and relatively easy to adapt to various growing conditions. They are nutritious and well-known for their distinctive flavors. In addition, many cole crops are rich in vitamin C, vitamin K, beta-carotene, and glucosinolates. These features make them potentially the ideal choices for space exploration. Like other *Brassica* family members, radishes are hardy plants as well. The variety we chose for this study showed shortened root length and total seedling length, indicating this variety may be more sensitive to space environment factors. Therefore, different varieties may have different responses to the combined space environment factors, which need to be evaluated further. Among three non-*Brassica* family plants, both tomato and lettuce showed reduced seedling growth. The immediate post-flight germination test we reported here has limitations for evaluating morphological changes. We used only water and light without substrates and nutrients. More grow-out tests using different growth conditions are ongoing at different post-flight storage time points to better characterize the changes induced by space exposure.

Temperature fluctuation is one of the major factors (in addition to space radiation, vacuum, and others) of the MISSE-FF that could also affect seed quality, resulting in the differences in root length and overall seedling length detected from this study. Compared to the ambient temperature at 22 °C for the ground controls, the average temperature for the flight samples was about 11 °C, derived from thermal data collected during each 90 min orbit, including a period of seven days at −40 °C. Because the iButtons are only able to record to −40 °C, temperatures may have been lower than −40 °C. To better interpret the data, more comparable ground controls are critical for this and future MISSE-FF studies.

As part of the validation test, ground control seeds were further exposed to comparable doses at ground-based simulated space radiation facilities (e.g. NSRL). Additionally, non-irradiated ground control seeds and irradiated ground control seeds will be exposed to thermal vacuum conditions simulating the ISS temperature profile (Fig. 4a) in a Thermal Vacuum Chamber located in the Microgravity Simulation Support Facility (MSSF) at NASA Kennedy Space Center. The capability to expose ground control samples to these high-fidelity space simulation chambers will provide more meaningful data and interpretation for these types of space exposure experiments using the MISSE-FF. All these validations deliver a future pathway to not only conduct meaningful MISSE-FF studies but also enable deep space science investigations, such as future experiments designed for cis-lunar orbit exposure and lunar surface missions.

Even though several experiments have previously flown plant seeds in space^10,11,21,22^, the radiation exposures reported in most of these published studies are not comparable to deep space radiation in quality, quantity, or both. Furthermore, many of these experiments (including the MISSE-Seed experiment reported here) are short-duration. Nevertheless, the radiation field on the ISS is very different from both the unshielded LEO radiation field and deep space radiation fields. Therefore, whether crop seeds, plant growth, and produce quality are significantly affected by long-term exposure to deep space radiation has yet to be adequately investigated and characterized. The limited capabilities of Earth-based radiation facilities to simulate space radiation scenarios contribute to this knowledge gap. Finally, extended stays in space will require the ability to cultivate food plants over multiple generations. As such, more needs to be learned about plant growth and reproduction in space environments. At present, little is known concerning the effects of ionizing radiation on plant reproduction, although studies in Chernobyl suggest that pollen viability is highly sensitive^23^, and this may have cascading effects on fruit production and seed germination^19^.

As a conclusion, the MISSE-Seed passive exposure platform was successfully validated to provide shielding from UV exposure and improved thermal exposure range for biological samples. The average exposure of ionizing radiation was 0.9 mSv/day (including about 93 days inside the ISS). Active MISSE-FF cell culture capability may be achievable by applying the concepts validated in this project to mitigate temperature fluctuations and adding active solar heating systems.

Eleven seed types survived the space environment for 338 days (with more than 245 days outside the ISS). These MISSE seeds germinated the same as ground control seeds, suggesting that the MISSE-FF exposure in the MPECs outside the ISS had no immediate effect on seed viability. Therefore, seeds are likely viable during deep space travel. Some species or plant varieties grew at different rates after the exposure than ground controls, suggesting that space environment exposure might affect seed quality and seed’s ability to respond to other environmental challenges. Several space environmental conditions, such as temperature fluctuations and space radiation, may have contributed to these changes. Ground-based validation experiments using the KSC thermal vacuum chamber and NSRL space radiation exposures will further illustrate the root cause of the changes observed in the seeds. Transcriptional regulation analyses are planned to target the underlying biological pathways.

This experiment and other studies cited here have shown that with proper shielding, plant seeds survive external exposure on the ISS to a very high extent, further proving the suitability and adaptability of plants in space travel. Future research should focus on using strategies at the molecular level to investigate survival mechanisms and genetic integrity. Another critical research focus is how space exposure affects plant’s abilities to respond to other environmental stresses, such as drought, overwatering, high CO_2_ exposure, pathogens, and many others that plants will likely encounter in space.

All these efforts will provide valuable baseline data, contribute to the design of deep-space food production systems for seed storage and radiation shielding, and identify candidate traits for successful plant growth during deep-space missions.

## Methods

### Seed selection

A complete list of these seed types is presented in Table 2. Most of these species or cultivars have been grown successfully under spaceflight-like conditions (temperature, CO_2_, humidity, lighting, etc.) using ground analogs at Kennedy Space Center (KSC). Some have also been grown on the ISS for science and crew consumption.

**Table 2:** List of crop seed types for pre-flight storage temperature test

Cultivar	Vendor	Product Number	Species
Outredgeous Lettuce	Johnny’s Selected Seeds	2208 N	*Lactuca sativa*
Dragoon Romaine Lettuce MT0 OG	Johnny’s Selected Seeds	3884 G	*Lactuca sativa*
Waldmann’s Dark Green Lettuce MT0 OG	Johnny’s Selected Seeds	428 G	*Lactuca sativa*
Shungiku	Johnny’s Selected Seeds	514	*Glebionis coronaria*
Toscano Kale	Johnny’s Selected Seeds	2123	*Brassica oleracea*
Red Russian Kale	Johnny’s Selected Seeds	363	*Brassica napus*
Extra Dwarf Pak Choi	Kitazawa Seed Co.	N/A	*Brassica rapa subsp. chinensis*
Dwarf Cauliflower Baby F1	Pure Line	N/A	*Brassica oleracea*
Mizuna Mustard	Burpee	50583 A	*Brassica rapa var. nipposinica*
Wasabi Arugula	Johnny’s Selected Seeds	3865 M	*Diplotaxis erucoides*
Amara Mustard	Johnny’s Selected Seeds	3147	*Brassica carinata*
Red Hybrid Pac Choi F1	Johnny’s Selected Seeds	3168	*Brassica rapa var. chinensis*
Scarlet Frills Mustard	Johnny’s Selected Seeds	2530	*Brassica juncea*
Hybrid Mini Broccoli BC1611 F1	Johnny’s Selected Seeds	4124	*Brassica oleracea*
Red Rambo Radish	Johnny’s Selected Seeds	2539 M	*Raphanus sativus*
Daikon Long Radish	Burpee	68030 A	*Raphanus raphanistrum*
Rosie Hybrid Pac Choi F1	Johnny’s Selected Seeds	3159	*Brassica rapa var. chinensis*
Garnet Giant Mustard	Johnny’s Selected Seeds	2797	*Brassica juncea*
Red Kingdom Mustard F1	Johnny’s Selected Seeds	3748	*Brassica rapa*
Tokyo Bekana Asian Cabbage	Johnny’s Selected Seeds	2251	*Brassica rapa var. chinensis*
Red Robin Tomato	Totally Tomatoes	612	*Solanum lycopersicum*
Bulgarian Carrot	Sandia Seed	N/A	*Capsicum annuum*
Trinidad Scorpion Pepper	Rare Seeds	N/A	*Capsicum chinense*
Fatalii Heirloom Pepper	Superhotchilies	N/A	*Capsicum chinense*
NuMex Heritage 6-4 Pepper	Sandia Seed	N/A	*Capsicum annuum*
Lumbre X-Hot Hatch Pepper	Sandia Seed	N/A	*Capsicum annuum*
Buckwheat	True Market	N/A	*Fagopyrum esculentum*
Barase Swiss Chard	Johnny’s Selected Seeds	N/A	*Beta vulgaris*
Bright Lights Swiss Chard	Johnny’s Selected Seeds	703 D	*Beta vulgaris*
Green Sorrel	Johnny’s Selected Seeds	383	*Rumex acetosa*
Black Oil Sunflower	True Market	N/A	*Helianthus annuus*

### Test of seed sensitivity to storage temperature

Seeds of these 32 seed types were separately sealed in individual 2.5 × 6.5 cm Mylar Foil Bags (IMPAK Corporation, Sebastian, FL USA) and continuously exposed to one of the five temperature conditions: −20 °C, −5 °C, 4 °C, 20 °C, and 38 °C for 21 days. At the end of temperature exposures, 10 seeds from each seed type and each storage condition were used for germination tests to determine seed sensitivity to the storage conditions. Even though the published historical temperature range for exposing biological organisms outside the ISS is −27 °C to 46 °C, the actual temperature fluctuations for each flight experiment are different and unpredictable. We used −20 °C, −5 °C, and 38 °C to test the potential temperature impact on seeds. The 21-day exposure timeframe was calculated based on an estimate of a 10 min exposure to the temperature conditions within each 90 min cycle for 6 months.

### Pre-flight germination test

Germination and pre-flight validation tests were conducted for all seed types following storage at different temperatures and before science integration. Briefly, 10 seeds were added from each temperature treatment to a 100 mm × 100 mm petri plate containing wetted germination paper. Petri plates were then sealed with parafilm and placed in a controlled environment chamber with growing conditions at 22 °C, 40–45% RH, 150 μmol m^−^^2^ s^−1^ light, and 16 h/8 h photoperiod. For pre-flight validation tests, 60 seeds per seed type were evaluated. Germination rates and developmental morphologies were documented.

### Final selection of seed types for the MISSE-seed experiment

Down selections to the final seed set for the MISSE-Seed experiment were derived from the germination tests following storage at different temperatures (Table 3). Other considerations were the seed size and whether seed types were identified as model organisms and used for other studies relevant to space exposure, such as exposures to radiation and ISS spaceflight. These seed types included: (1) The model plant, *Arabidopsis thaliana*, which has been studied extensively on the ISS^24,25^; (2) crop species/cultivars that had been grown on the ISS (amara mustard, mizuna, lettuce, and radish)^26–28^; (3) crop species/cultivars that have been tested extensively under simulated ISS condition by the KSC Space Crop Production team (chili peppers, tomato, cauliflower, pac choi, Garnet Giant mustard, and Scarlet frills mustard). *Arabidopsis*, mizuna, lettuce, and tomato seeds were also used for a KSC seed radiation study conducted in the NSRL to investigate the impact of simulated galactic cosmic rays (GCR) and solar particle event (SPE)^17,29^.

**Table 3:** Down-selected seeds used for the MISSE-seed flight experiment

	Cultivar	Species	Vendor	Product ID	Germination Rate (%)	Number of Packets	
						Flight	Ground control
1	Outredgeous Lettuce	*Lactuca sativa*	Johnny’s Selected Seeds	2208 N	98.3	12	12
2	Baby F1 Cauliflower	*Brassica oleracea var. botrytis*	Pure Line Seeds	N/A	96.7	12	12
3	Mizuna Mustard	*Brassica rapa var. nipposinica*	Burpee	50583 A	98.3	12	12
4	Amara Mustard	*Brassica carinata*	Johnny’s Selected Seeds	3147	95	12	12
5	Scarlet Frills Mustard	*Brassica juncea*	Johnny’s Selected Seeds	2530 M	86.7	12	12
6	Red Rambo Radish	*Raphanus sativus*	Johnny’s Selected Seeds	2539 M	90	12	12
7	Rosie Pac Choi	*Brassica rapa var. chinensis*	Johnny’s Selected Seeds	3159 M	91.7	12	12
8	Garnet Giant Mustard	*Brassica juncea*	Johnny’s Selected Seeds	2797 M	93.3	12	12
9	Red Robin Tomato	*Solanum lycopersicum L*.	Totally Tomatoes	612	90	12	12
10	NuMex 6-4 Pepper	*Capsicum annuum*	Sandia Seed	N/A	80	12	12
11	At Col-0 Varity	*Arabidopsis thaliana*	In-house	N/A	90	20	20

### MPEC assembly and environmental data collection

Prior to MPEC science integration, germination rates were confirmed for each seed type (Table 3). Twenty-five to 30 seeds from each crop seed type and about 1000 *Arabidopsis* seeds were packed within individual Mylar foil bags, heat sealed, and kept at 4 °C through the pre-flight sample preparation process. Twelve to 34 seed packets were then integrated into each MPEC depending on the MPEC configuration.

In addition, thermoluminescent dosimeters (TLDs) and iButton temperature dataloggers (DS1922L-F5# Thermochron iButton 8 K, iButtonLink Technology, Whitewater, WI 53190 USA) were included inside each MPEC for documenting environmental conditions. Temperature loggers were programmed to record data every 10 min (in duplicate) in order to capture a reliable thermal profile throughout the 90 min ISS orbital cycles. TLDs included three types: 100, 600, and 700 (one of each for each container), which were calibrated using SPEsim exposures in the NSRL. These TLDs capture different aspects of the radiation spectrum and were used to validate the measurements. An additional set of ground control MPECs using the same configuration was integrated for the Ground Control set of seeds.

A pre-flight validation test was conducted to evaluate whether the sealed Mylar packets and data-loggers could sustain vacuum conditions. The results are presented in the “Pre-flight validation test results” section.

### Science turnover and flight implementation

**Fig. 6:**
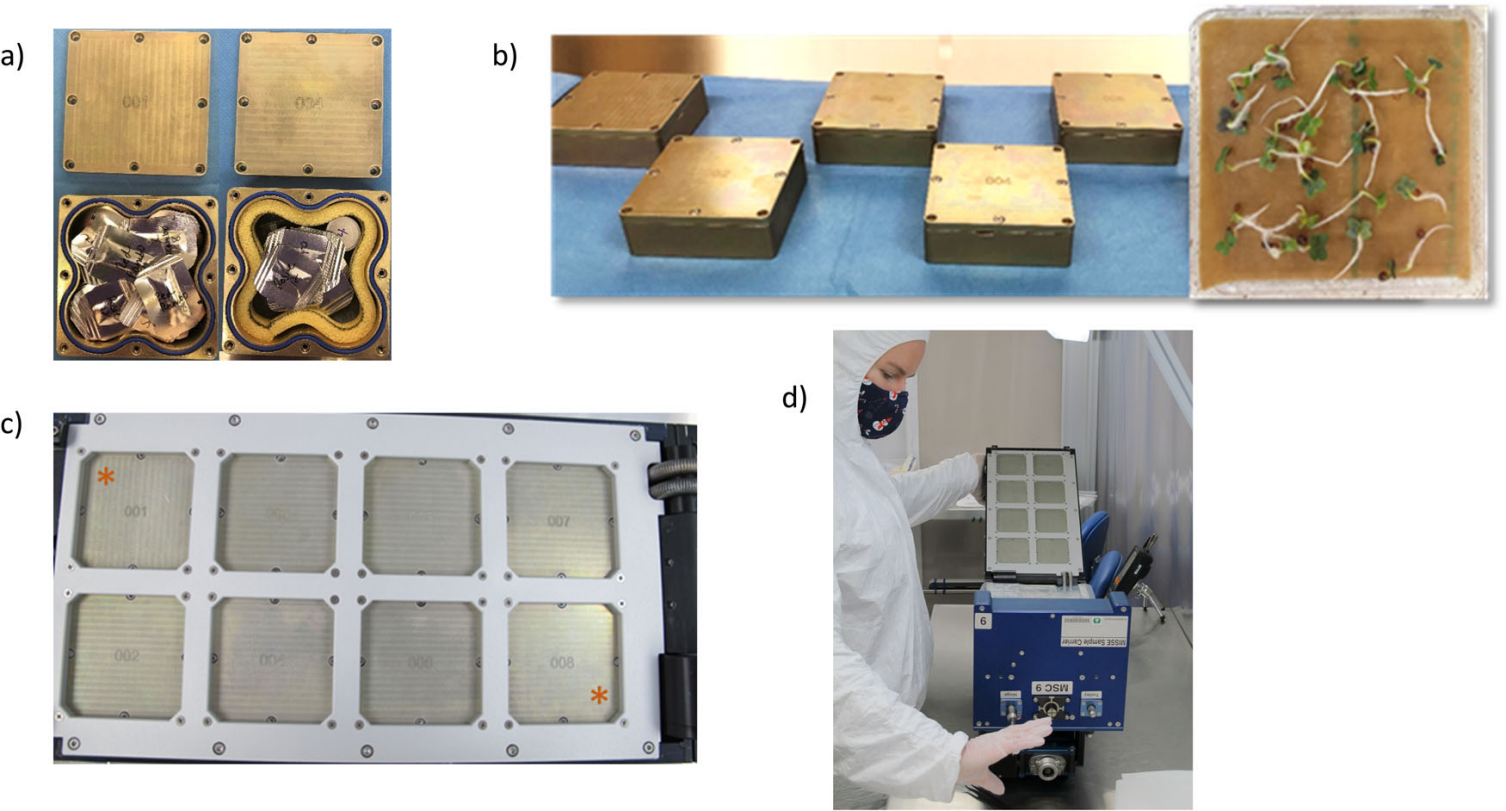
Science integration. **a** Example flight MPECs with or without insulation foams. **b** Fully assembled MPECs and an example of pre-assembly germination tests (mizuna). **c** The MISSE-Seed Panel with eight flight MPECs (* indicating the MPECs without insulation materials). **d** The MISSE-Seed Panel assembled with the MSC at the swing side and tested in the Aegis facility before the NG-15 launch.

Eight MPECs were assembled for flight, and eight identical units integrated for Ground Control were assembled five months later to simulate flight conditions (Fig. 6a, b). Six MPECs included thermal insulation materials, and two did not which were marked * in Fig. 6c. The containers were shipped at room temperature (RT) to the Aegis Aerospace MISSE-FF facility (Houston, TX) and maintained at ambient temperatures during integration into the MISSE-FF, during transit to ISS onboard NG spacecraft, inside the ISS, and during sample return. The MPECs were then installed onto the MSC panel at the swing side in the MISSE-FF (Fig. 6c). The MSC also provided sensors for recording UV fluence rates, radiation dose, atomic oxygen levels, and temperatures after the MSC opening.

### Flight operations

**Fig. 7:**
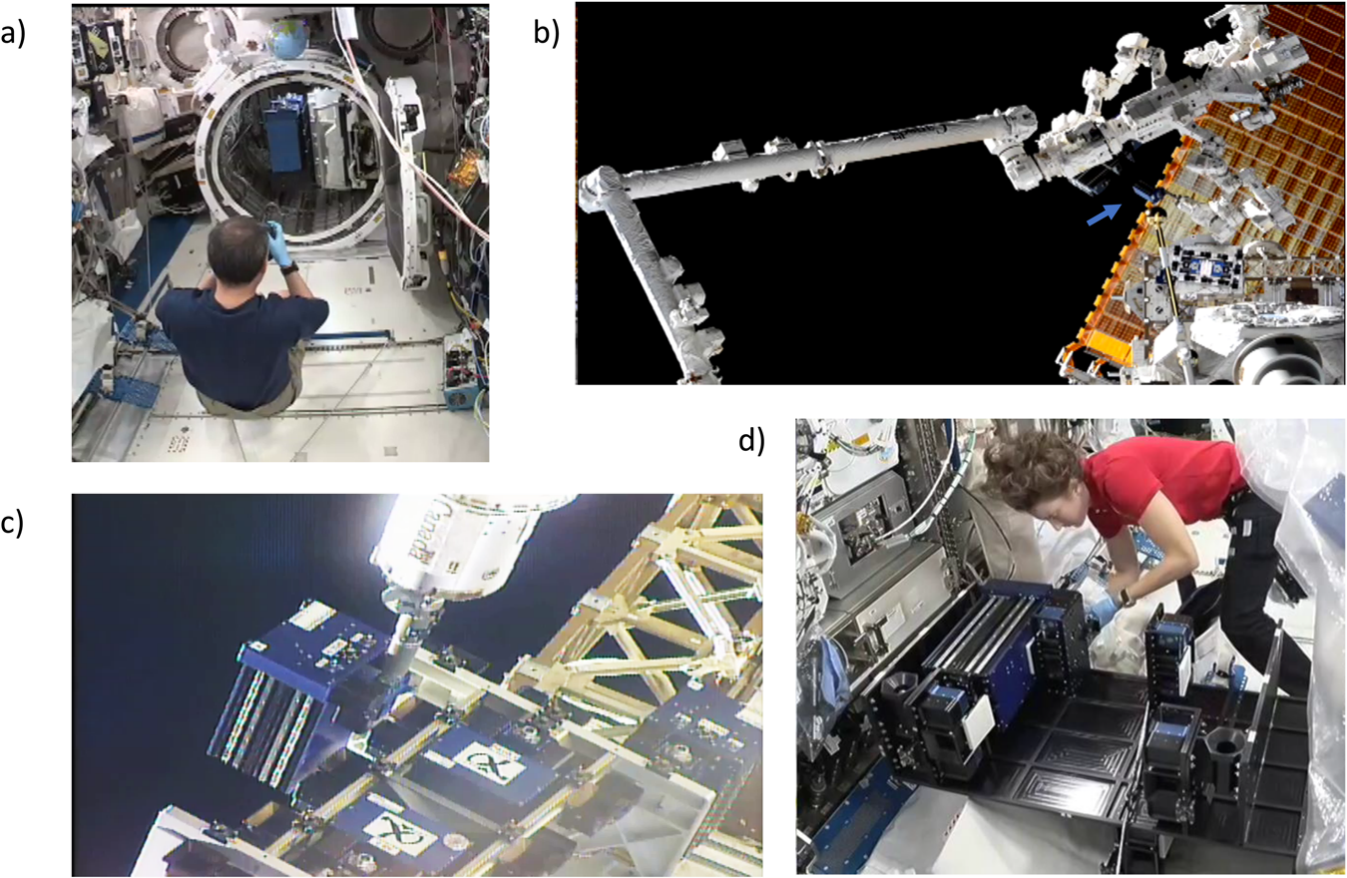
Flight operations. **a** MISSE-14 inside the JEM airlock; **b**, **c** MISSE-14 installation outside the ISS (blue arrow pointing to the MISSE-14 payload); and **d** MSCs packaged for return. All photos courtesy of NASA/the ISS Program Office.

The MISSE-Seed experiment was launched to the ISS aboard NG-15 on 02/20/21 as part of the MISSE-14 mission which consisted of an 8-month exposure to the space environment outside ISS (Fig. 7). The MISSE-Seed panel was mounted on the swing side of the MSC at the MISSE-FF Zenith position facing deep space (Fig. 7). MISSE-14 MSCs were installed on the MISSE-FF outside ISS between 04/21/21 and 04/25/21. MSCs were exposed to the environment outside ISS in an unopened configuration for two months then opened on 06/21/21 and exposed to the environment outside ISS in an opened configuration for six months. After exposure, they were moved from MISSE-FF to the JEM Airlock inside ISS between 12/26/21 and 12/29/21. MISSE-Seed specimens were returned to Earth at nominal temperature on SpX-24 on 01/23/22.

### Post-flight analysis

After samples were returned from the ISS, MPECs were disassembled, and seed packets were stored at 4 °C until testing for seed viability and seedling development using the same protocol and growing conditions as the pre-flight tests. Seeds from each variety were planted in three Petri plates with 5–7 seeds planted on each plate for crop varieties and 15–56 seeds on each plate for *Arabidopsis*. At 6–13 days after planting (durations were species dependent), seedlings were harvested for assessment of germination rates, morphological changes, and seedling length measurements. Images were acquired for evaluating signs of stress and developmental abnormalities. Total seedling and root lengths were measured using forceps and rulers. Data were presented as mean ± SEM from three replicate plates for germination rate or from the total number of seedlings for seedling/root length measurements. Statistical analysis was performed using a student’s *t*-test.

## Data Availability

The data generated by the KSC MISSE-Seed team are available from Y.Z. upon request.
